# 

**Published:** 1908-05-09

**Authors:** 


					hi a u ?* l;:? *
SUHGtOJ* G??'> RA-_ -irr I
MM.- i i ??h:j
Telegraphic Address i THE MODERN^tjTelephone Number :
Oopednle, London. MEDICAL JOURNAL?. ? 1 G.rr.rd.
HEW SERIES. No. 60, Vol. III. SATURDAY
No. 1132. Vol. XLIV. oATUKLJAx,
May 9th, 1908.
PRICE THREEPENCE.

				

